# Impact of COVID-19 on the thyroid gland: an update

**DOI:** 10.1007/s11154-020-09615-z

**Published:** 2020-11-25

**Authors:** Lorenzo Scappaticcio, Fabián Pitoia, Katherine Esposito, Arnoldo Piccardo, Pierpaolo Trimboli

**Affiliations:** 1 Division of Endocrinology and Metabolic Diseases, University Hospital “Luigi Vanvitelli”, University of Campania “L. Vanvitelli”, Naples, Italy; 2 Division of Endocrinology, Hospital de Clínicas, University of Buenos Aires, Buenos Aires, Argentina; 3 Department of Advanced Medical and Surgical Sciences, University of Campania “L. Vanvitelli”, Naples, Italy; 4 Diabetes Unit, University Hospital “Luigi Vanvitelli”, University of Campania “L. Vanvitelli”, Naples, Italy; 5 Department of Nuclear Medicine, Galliera Hospital, Genoa, Italy; 6 Clinic of Endocrinology and Diabetology, Lugano and Mendrisio Regional Hospital, Ente Ospedaliero Cantonale, Bellinzona, Switzerland; 7 Faculty of Biomedical Sciences, Università della Svizzera Italiana (USI), Lugano, Switzerland

**Keywords:** Thyroid, COVID-19, Hyperthyroidism, Hypothyroidism, Thyroid cancer

## Abstract

Coronavirus disease 2019 (COVID-19) is the pandemic of the new millennium. COVID-19 can cause both pulmonary and systemic inflammation, potentially determining multi-organ dysfunction. Data on the relationship between COVID-19 and thyroid have been emerging, and rapidly increasing since March 2020. The thyroid gland and the virus infection with its associated inflammatory-immune responses are known to be engaged in complex interplay. SARS-CoV-2 uses ACE2 combined with the transmembrane protease serine 2 (TMPRSS2) as the key molecular complex to infect the host cells. Interestingly, ACE2 and TMPRSS2 expression levels are high in the thyroid gland and more than in the lungs. Our literature search provided greater evidence that the thyroid gland and the entire hypothalamic–pituitary–thyroid (HPT) axis could be relevant targets of damage by SARS-CoV-2. Specifically, COVID-19-related thyroid disorders include thyrotoxicosis, hypothyroidism, as well as nonthyroidal illness syndrome. Moreover, we noticed that treatment plans for thyroid cancer are considerably changing in the direction of more teleconsultations and less diagnostic and therapeutical procedures. The current review includes findings that could be changed soon by new results on the topic, considering the rapidity of worldwide research on COVID-19.

## Introduction

Coronavirus disease 2019 (COVID-19) is the pandemic of the new millennium with unprecedented issues for global health [1]. The causative agent is a novel enveloped RNA β-coronavirus 2 that has been named severe acute respiratory syndrome coronavirus 2 (SARS-CoV-2) [2]. Since it was first identified in Wuhan, COVID-19 is spreading rapidly, and outbreaks are growing at an exponential rate [3]. As of 16 August 2020, the number of patients infected with SARS-Cov-2 has exceeded 21.294.845 globally, and more than 761,700 persons have now died from COVID-19 [3]. SARS-Cov-2 has a phylogenetic similarity to SARS-CoV-1, the virus responsible for the severe acute respiratory syndrome (SARS) [2, 4, 5]. Similar to SARS-CoV-1, SARS-CoV-2 infects human tissues entering cells through the angiotensin-converting–enzyme 2 (ACE2) receptor [4, 5].

Coronaviruses infection has a wide spectrum of clinical severity, ranging from asymptomatic cases and the common cold to more severe and even fatal respiratory damage [6]. SARS-CoV-2 infection can cause both pulmonary and systemic inflammation, determining multi-organ dysfunction in patients with high risk factors (i.e. old age, male gender, chronic hypertension and other cardiovascular comorbidities, diabetes) [7, 8]. Acute respiratory distress syndrome (ARDS) and respiratory failure, sepsis, acute cardiac injury, and heart failure are considered the most common critical complications of COVID-19 [7].

Both direct (i.e. caused by the virus infection of the target cells) and indirect injury (i.e. through abnormal immune-inflammatory responses to the virus and likely involving the coagulation, cytokine and complement systems) have been linked to the wide clinical expression spectrum and multisystem organ failure of COVID-19 and SARS [9–13].

Nowadays, the response from the worldwide research community to win the COVID-19 pandemic fight has been vigorous, and a multitude of studies regarding the varying aspects of the disease (i.e. prevention, diagnosis, and therapy) have been carried out and the results will be published in the near future [14]. Nonetheless, data on the relationship between COVID-19 and thyroid have been emerging, and rapidly increasing since March 2020 [15].

The thyroid gland and the virus infection are known to be engaged in complex interplay via hormones and immunomodulatory signaling molecules [16, 17]. These connections have been established in physiological and pathological settings [16, 17]. Viruses with its associated inflammatory-immune responses could be regarded as a major variable which might affect lifelong thyroid function, consequently contributing to define the “thyroid biography” at the individual level [18].

Thyroid hormones modulate innate and adaptive immune responses through both genomic and nongenomic mechanisms [16]. Physiological concentrations of L-thyroxine (T4) and 3,3^’^,5-triiodo-L-thyronine (T3) stimulate the production and release of cytokines, which are also components of “cytokine storm” potentially characterizing systemic viral infections [19, 20]. Moreover, thyroid hormones are capable to potentiate the antiviral action of IFN-γ [16]. It is also of interest that some pathways (i.e. the cytokine and hyperactivation of Th1 helper cells responses) of immune responses to virus infection are observed in thyroid disorders [i.e classical autoimmune thyroid diseases (AITD), interferon-alpha-related thyroid disease, immune checkpoint inhibitor mediated thyroiditis, alemtuzumab-induced thyroid dysfunctions] [17, 21–24]. Yet, clinicians are very familiar with the evidence that infection can be identified as an environmental stimulus precipitating or accelerating AITD development and the cause of subacute thyroiditis [17, 25]. On the other hand, respiratory infections could potentially precipitate a thyroid storm in patients with decompensated hyperthyroidism, which in turn may favour the infection-related mortality risk due to cardiovascular morbidity [26]. It is also important to note that T4 is known to activate human platelets [27] and this could sustain pathological clotting encountered as a complication of virus infections. These and other remarks warrant an improved knowledge of the relationship between COVID-19 and thyroid.

We conducted a comprehensive search of PubMed and MEDLINE articles using the combination of the search terms “thyroid” and “coronavirus” (or “SARS-CoV-2” or “COVID-19”) with no limits on date and no language restrictions. As of 5 Sempteber 2020, the search strategy showed 105 articles. This was complemented by a carefully hand-searching reference lists for additional studies. Most of the published studies were a collection of expert opinions and recommendations on the new strategies of care of thyroid patients in the face of COVID-19 transmission risk and health care surge capacity [8, 28–34]. Instead, only 13 articles explored thyroid function and/or reported new-onset thyroid diseases in patients contracting COVID-19 [7, 35–46]. Only two studies investigated the histopathological characteristics of the thyroid gland and viral thyroid tropism from patients who died of severe COVID-19 [47, 48].

What we review next are the new findings on SARS-CoV-2 infection and thyroid. Specifically, first, we will resume the basis for a relationship between COVID-19 and the thyroid gland; then, we will examine the COVID-19 related thyroid disorders that emerged to date; and lastly, we will address the data regarding the clinical experiences in thyroid cancer patients’ care during the COVID-19 pandemic.

## Thyroid and COVID-19

As already mentioned, both SARS-CoV-1 and SARS-CoV-2 use ACE2 combined with the transmembrane protease serine 2 (TMPRSS2) as the key molecular complex to enter and infect the host cells [4, 5]. Interestingly, ACE2 and TMPRSS2 expression levels are high in the thyroid gland and more than in the lungs [5, 49, 50]. The *in silico* approach also shows that in the thyroid ACE2 expression levels are positively and negatively linked to immune signatures [i.e. CD8^+^ T cells, interferon response, B cells, and natural killer (NK) cells] in males and females, respectively, [50] thus contributing to explain the different immune responses and the resultant distinct thyroid manifestations. Uptake by host cells of SARS-CoV-2 is thought secondarily to involve other cellular molecules and proteases [4, 5]. One main group of structural proteins of the plasma membrane that could be implicated in the cell invasion of SARS-CoV-2 is represented by integrins [51]. ACE2 binds to integrin to modulate downstream signal transduction [51]. Herein, it is worth noting that T4 regulates expression of the genes for the monomeric protein that makes up integrins and thyroid hormones are deemed to promote internalization of the integrins [52, 53]. Therefore, thyroid hormones could positively influence the SARS-CoV-2 uptake involving integrins [53].

As for ACE2 and TMPRSS2, a peripheral expression of olfactory receptors (ORs) has been demonstrated, including a wide expression profile at the thyroid gland [54]. The impairment of ORs signaling/function in the nasal neuro-epithelium or the olfactory bulb constitutes the molecular mechanism that underlies the loss of smell (anosmia) in patients with COVID‑19 [55]. Since ORs are co-expressed with the key mediators of SARS-CoV-2 cell entry (i.e.ACE2, TMPRSS2, cathepsin L) it has been postulated that their damage could be involved in sequelae of COVID-19 from other peripheral organs, not excluding the thyroid [54]. Moreover, SARS-CoV-2 could also indirectly affect the thyroid gland, since “hyperactivity of Th1/Th17 immune responses” and “cytokine storm” associated to COVID-19 may trigger and perpetuate the thyroid gland inflammation [42].

Anatomopathological studies on patients with SARS as cause of death were previously performed to describe the histopathological findings in the thyroid gland [56–58]. Ding et al. [56] did not detect viral genomic sequences in the thyroid, while Gu et al. [57] found SARS genomic sequence positive lymphocytes and monocytes in the vessel of the thyroid gland from a SARS autopsy, along with no obvious pathologic changes. It is of note that the infection of immune cells could sustain the hypothesis of virus dissemination across different systems of the body outside the respiratory system. Instead, an extensive injury to the follicular epithelium and parafollicular cells was described by Wei et al. [58] in thyroid tissue specimens of all five patients who died of SARS. As stated by the authors [58] the observed changes in these SARS thyroid glands could be consistent with apoptosis, since neither inflammatory infiltrates nor morphological cellular features of necrosis were identified at the microscopic examination. Normal function of calcitonin consists of inhibiting the osteolysis and increasing calcium deposition in the bone. A captivating explanation of the increased risk of osteonecrosis of the femoral head (ONFH) among both SARS and COVID-19 patients could be the extensive damage to the parafollicular cells, beyond the negative effects of the improper use of corticosteroid-based SARS and COVID-19 treatment [58, 59].

However, it cannot be excluded that SARS-related thyroid damage may be secondary to a hypothalamic-pituitary system virus insult leading to thyroid disconnection. In this regard, SARS genome sequences were detected in the cytoplasm of numerous neurons in the hypothalamus [57] and the immunohistochemistry evaluation of adenohypophysis from autopsies of five SARS patients revealed that both the number and the immunoreactive intensity of TSH positive cells were markedly decreased [60].

The histopathological findings of the thyroid gland in patients with SARS-CoV-2 infection have been published in only two studies [47, 48]. Both studies [47, 48] reported lymphocytic infiltration in the interstitium in three of three patients and in two of nine patients, respectively. In the two patients of Hanley et al. [48] follicular epithelial cell disruption was also noted. However, the significance of these histopathological data regarding the thyroid gland in patients with COVID-19 is uncertain.

**Fig. 1:**
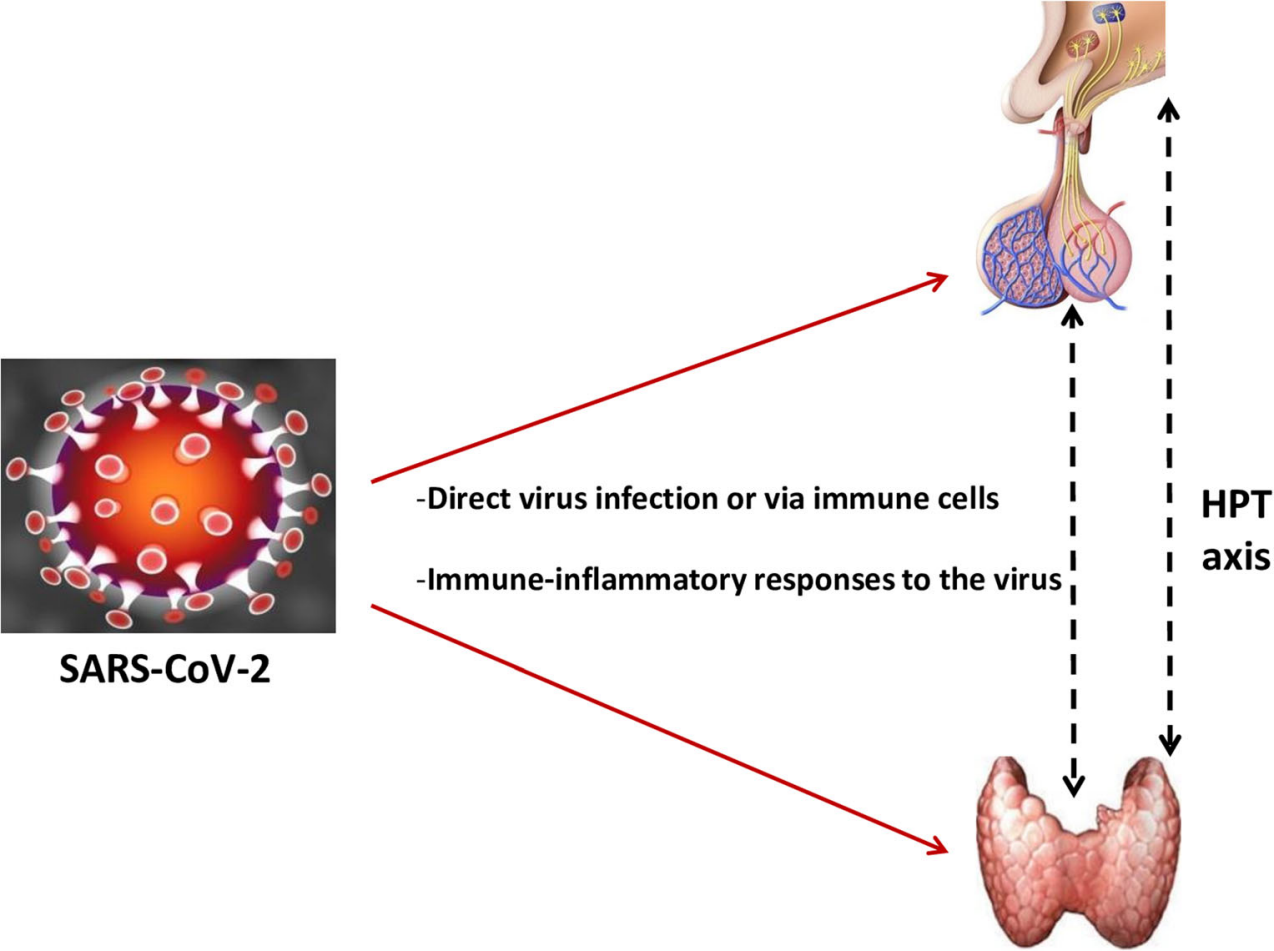
Schematic representing potential mechanisms of hypothalamic–pituitary–thyroid (HPT) axis injury by SARS-CoV-2 infection

Broadly, two plausible mechanisms might account for the changes in the thyroid gland and its hypothalamic–pituitary axis. One is an indirect effect through abnormal systemic inflammatory-immune responses caused by SARS-CoV-2 infection, and another is a direct viral effect (Fig. 1).

## Thyroid dysfunction in patients with COVID-19

Assessment of thyroid function for COVID-19 is not recommended by the World Health Organization clinical management guidelines [61]. Nevertheless, during the previous coronavirus outbreak with SARS-CoV changes in thyroid function were already observed in some studies [62, 63]. In particular, the study by Wang et al. [62] reported that serum levels of TSH, T3 and T4 in patients with SARS-CoV were significantly lower than those in the control group. They found [62] a positive correlation between the severity of SARS and levels of T3, so that the more severe the disease the lower was the level of T3. Moreover, different figures of low levels of thyroid hormones were found according to the phase of disease: T3 and T4 levels were decreased, respectively, in 94% and 46% of patients during the acute phase and in 90% and 38% during the convalescent phase [62]. Similarly, Leow et al. [63] reported that four (6.7%) SARS patients three months following recovery were biochemically hypothyroid, comprising three with central hypothyroidism and one with primary hypothyroidism due to new-onset chronic lymphocytic thyroiditis. While central hypothyroidism spontaneously remitted in the three patients with central hypothyroidism after three/nine months, the case with primary hypothyroidism required permanent T4 therapy [63].

Therefore, from the SARS epidemic, we learned that the virus infection could mainly produce low thyroid function caused by a primary thyroid injury or a secondary injury (i.e. at hypothalamic or pituitary level), either alone or in combination, permanent or transitory. In addition, notably with regard to severe or critically ill patients, the low levels of TSH and T3 could be considered as part of the adaptive state of “nonthyroidal illness syndrome” triggered by a major stressful situation (i.e. the systemic virus disease). These hormonal changes could be explained by the above described histopathological findings of the virus infection at the thyroid and/or the hypothalamic-pituitary sites [57, 58, 60].

**Fig. 2:**
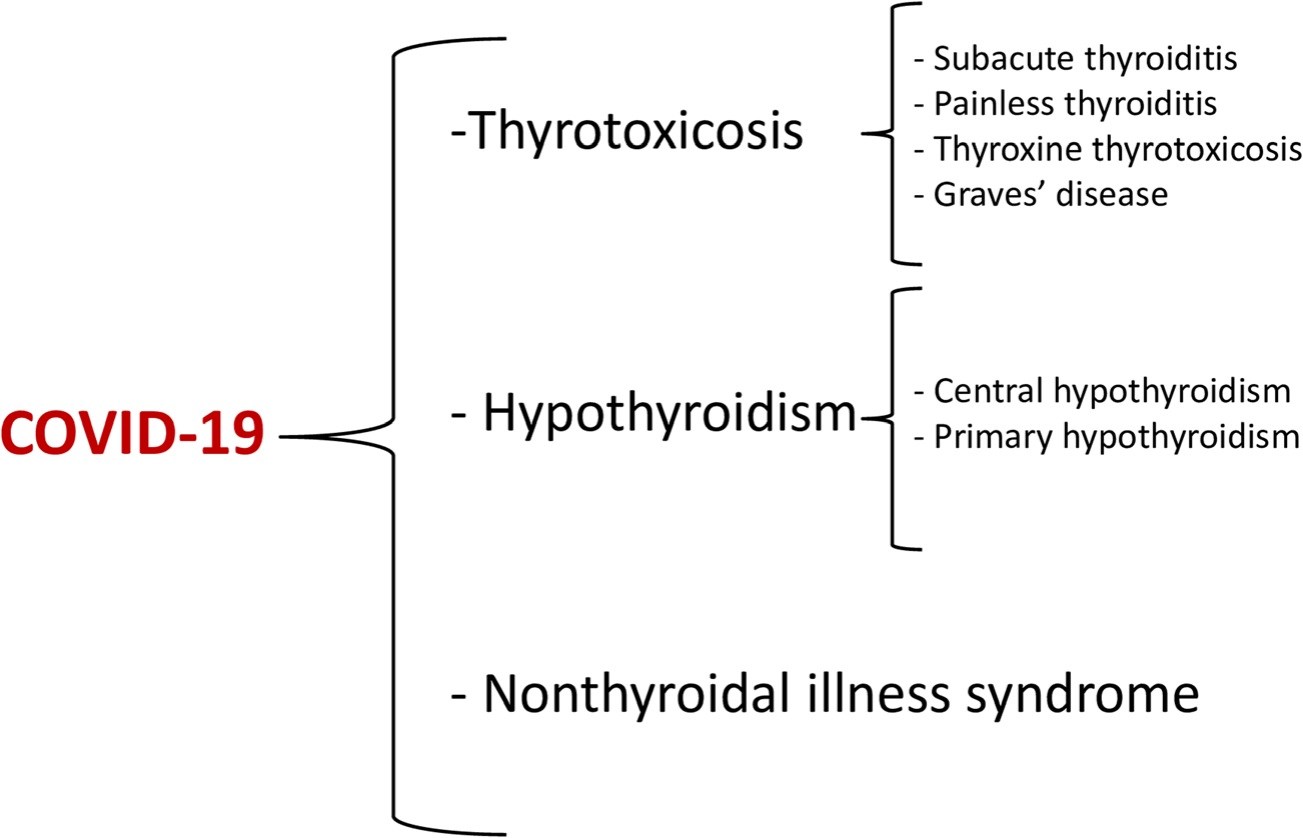
Covid-19-related thyroid disorders emerged from the analysis of the current literature

Furthermore, our literature search relative to thyroid dysfunction in patients with COVID-19 provided greater evidence that the thyroid gland and the entire hypothalamic–pituitary–thyroid (HPT) axis could be emerging and relevant targets of damage by SARS-CoV-2. Specifically, COVID-19-related thyroid disorders could biochemically manifest as thyrotoxicosis, hypothyroidism, as well as nonthyroidal illness syndrome (Fig. 2).

### Thyrotoxicosis

Subacute thyroiditis (SAT) (also named De Quervain thyroiditis) is a self-limited thyroid disease caused by a viral or postviral inflammatory process [64]. Neck pain is the hallmark of the clinical syndrome, that’s why another synonym is “painful subacute thyroiditis” [64]. The clinical course of SAT usually shows three consecutive phases: first thyrotoxicosis during the first few months, followed by hypothyroidism for about three months and then by euthyroidism [64]. Many viruses are known to be associated with the development of SAT, and evidence for infection can be based on epidemiological, serological (or circulating viral genome) or direct evidence data [65]. Direct evidence of the presence of viruses or their components in the thyroid tissue is available only for few viruses [65]. Virus infections could be responsible for thyroid diseases by liberating antigens (via necrosis or apoptosis), by forming altered antigens or causing molecular mimicry, by proinflammatory cytokine and chemokine secretion, by inducing aberrant HLA-DR expression and Toll-Like Receptor (TLR) activation [65]. It was conceivable that also SARS-CoV-2 could be associated to SAT [66]. Table 1 collects the main features of the nine COVID-19-related SAT cases that have been reported to date. Patients were all females except one, and age ranged from 18 to 69 years, as expected in general for SAT outside of the COVID-19 pandemic [64]. Previous autoimmune thyroid disease or dysfunction was absent in all cases. Evidence of SARS-CoV-2 infection was demonstrated by the presence of viral RNA in oropharyngeal or nasopharyngeal swabs along with quantitative detection of serum specific IgG and IgM in three cases. Covid-19 manifestations were mild in eight of nine cases, while interstitial pneumonia affected the oldest patient with SAT (i.e. 69 years old). It is important to note that in six of nine patients (about 65%) SAT occurred after remission of COVID-19 (i.e. clinical disappearance and negative virus detection tests), with a time interval from COVID-19 ranging from 17 to 40 days. Conversely, in three cases [38, 39, 41] SAT presented along with manifestations of SARS-CoV-2 infection, at admission or during the first days of hospitalization. It is noteworthy that in the patient with SARS-CoV-2-related pneumonia [38] control swab test continued to be positive two months after the COVID-19 diagnosis. Neck pain (optionally radiated to the jaw and/or the ear) was present in eight of nine cases (about 90%), and it was missing only in the oldest patient with SARS-CoV-2-related pneumonia who was also on painkillers for previous back surgery [38]. Moreover, fever accompanied neck pain in five cases (about 60%). The degree of biochemical thyrotoxicosis could range from mild to moderate: indeed, maximum serum free T4 (FT4) and free T3 (FT3) levels could be about two times the upper limit of the normal. TSH receptor antibodies (TRAb) and thyroperoxidase (TPOAb) antibodies were negative in all cases, while thyroglobulin antibodies (TgAb) were positive in two patients of whom one needed T4 for subsequent hypothyroidism [36]. C-reactive protein (CRP) values were high in all cases and they could range from 8 to 122 mg/L. Manifestations of early-onset SAT could include different signs and symptoms such as goiter, fatigue, palpitations, inappetence, sweating, insomnia, anxiety, tremor, weight loss. Nevertheless, the 38-year-old female with no history of cardiovascular disease experienced atrial fibrillation [36]. In the context of SAT thyrotoxicosis, atrial fibrillation is rarely described [67], while this is one of the main arrhythmias resulting from the systemic inflammatory response and myocardial injury of COVID-19 [68]. Thus, it is conceivable that in patients with thyrotoxicosis and COVID-19 (current or recent past infection with SARS-CoV-2) atrial fibrillation could be due to both the hormonal excess and the systemic inflammatory response [36, 42]. In all cases, thyroid imaging (i.e. ultrasound or scintigraphy) features corresponded to that of classical SAT at the time of destructive thyrotoxicosis. Also as regards the therapeutical and outcome characteristics, COVID-19-related SAT was similar to SAT secondary to other viruses: in all cases, steroidal and non-steroidal anti‐inflammatory drugs (NSAIDs) were effective to obtain a quick resolution of thyrotoxicosis and normalization of inflammatory markers. Glucocorticoid use in patients with COVID-19 has been proven to be of benefit in selected cases [69]. Considering the potential cardiovascular complications of both COVID-19 and SAT thyrotoxicosis, a low dose regimen of steroids to treat SAT thyrotoxicosis and neck pain could positively impact on the outcome of patients with COVID-19-related SAT. Hypothyroidism after SAT occurred in only two cases [36], and relapse of COVID-19 (both clinically and at diagnostic tests) was excluded in six cases [35, 36, 40].

**Table 1:** Analysis of cases of COVID-19-related subacute thyroiditis (SAT) reported in the literature to date

*Case*, (ref.)	1, (35)	2, (36)	3, (36)	4, (36)	5, (36)	6, (38)	7, (39)	8, (40)	9, (41)
Sex	F	F	F	F	F	F	F	F	M
Age (yr)	18	38	29	29	46	69	41	43	34
Thyroid disease before Covid-19	no	no	no	no	no	nodules	no	no	no
Covid-19 test	swab	swab	swab, sIg	swab, sIg	swab	swab	swab	swab, sIg	swab
Covid-19 manifestations	mild	mild	mild	mild	mild	pneumonia	mild	mild	mild
Time from Covid-19 to SAT onset (days)	17	16	30	36	20	during Covid-19	during Covid-19	40	during Covid-19
Doctor’s visit	outpatient, in-person	outpatient, in-person	outpatient, in-person	outpatient, in-person	outpatient, in-person	inpatient	inpatient	outpatient, in-person	inpatient
SAT manifestations	typical, neck pain, fever (37.5 °C)	typical, neck pain, fever (38.5 °C), AF	typical, neck pain	typical, neck pain	typical, neck pain, fever (37.2 °C)	typical, no neck pain	typical, neck pain, fever (38.5 °C)	typical, neck pain, fever (37.5 °C)	typical, neck pain
Biochemical profile	TSH 0.004 FT4 27.2 FT3 8.7 TgAb+ TPOAb- TRAb-	TSH 0.1 FT4 29.3 FT3 8.0 TgAb- TPOAb- TRAb-	TSH 0.01 FT4 31.8 FT3 8.9 TgAb+ TPOAb- TRAb-	N.A.	TSH 0.01 FT4 27.8 FT3 6.9 TRAb-	TSH 0.08 FT4 31.6 FT3 7.0 TgAb- TPOAb- TRAb-	TSH 0.08 FT4 25.7 FT3 7.7 TgAb- TPOAb- TRAb-	TSH 0.006 FT4 34.6 FT3 9.0 TgAb- TPOAb- TRAb-	TSH 0.01 FT4 41.8 FT3 13.4 TPOAb- TRAb-
Inflammatory markers	WBC 11.2, CRP 6.9	CRP 11.2	CRP 7.9	N.A.	CRP 8	N.A.	WBC 15.6, CRP 101	WBC 6.6, CRP 8.8	WBC 11.6, CRP 122
Thyroid US features	typical	typical	typical	typical	typical	typical	typical	typical	typical
Thyroid scintigraphy uptake	N.A.	N.A.	absent	N.A.	N.A.	absent	N.A.	markedly reduced	N.A.
Resolutive therapy	prednisone	prednisone	prednisone, propanolol	ibuprofen	prednisone	prednisone	prednisolone	prednisone	prednisolone, atenolol
Thyroid function after SAT	normal	normal	hypothyroidism	hypothyroidism	normal	N.A.	N.A.	normal	normal
Relapse of Covid-19	no	no	no	no	no	swab+	N.A.	no	N.A.

Ref., reference; yr, years; US, ultrasound; F, female; TSH, thyrotropin; FT4, free thyroxine; FT3, free triiodothyronine;

TgAb, thyroglobulin antibodies; TPOAb, thyroperoxidase antibodies; TRAb, TSH receptor antibodies; WBC, white blood cells;

CRP, C-reactive protein; N.A., not available; AF, atrial fibrillation; sIg, serum immunoglobulin

TSH, FT4 and FT3 expressed as mIU/L, pmol/L and pmol/L, respectively. WBC as number x10^9^/L, CRP as mg/L

Swab was obtained from oropharyngeal or nasopharyngeal mucosa

A quantitative assay was used for the detection of serum SARS-CoV-2-specific IgG and IgM

Mild Covid-19 manifestations could include: fever, rhinorrhea, painful swallowing, cough, hoarseness, anosmia, conjunctivitis, asthenia, with speedy (some days, maximum 14 days) and complete recovery

Typical manifestations of SAT could include: goiter, fatigue, palpitations, inappetence, sweating, insomnia, anxiety, tremor, weight loss

Typical US features were consistent with hypoechoic areas and absent/low vascularization at color Doppler ± goiter

Thyroid scintigraphy was done with ^99m^Technetium

Caution needs to be given to the interpretation of the aetiology of the COVID-19-related SAT, since in all the nine cases SARS-CoV-2 was not directly evidenced in the thyroid tissue, but the proof of the virus infection was based on epidemiological and/or serological (or circulating viral genome) data [65]. However, because of the above, clinicians should know that SAT can occur during and after COVID-19. Neck pain, that can be mistaken for common sore throat of COVID-19, and persistent tachycardia (despite the clinical amelioration of COVID-19 and the absence of other common cardiac causes) should suggest COVID-19-related SAT.

In the study by Lania et al. [42] a high number of patients (58/287, 20.2%) hospitalized for COVID-19 in non-intensive care units was found to be affected by thyrotoxicosis in absence of neck pain, likely identifying patients with COVID-19-related painless (silent) thyroiditis (or more roughly destructive thyroiditis cases without neck pain). Overt thyrotoxicosis (i.e. defined as low TSH values with FT3 and/or FT4 above the reference ranges) was diagnosed in 31 of 58 patients with thyrotoxicosis (53.4%) and an inverse and robust relationship between serum TSH and IL-6 levels was recorded, supporting the hypothesis of an inflammatory-mediated damage to the thyroid gland [42]. The absence of neck pain and the TPOAb positivity are two main features of painless thyroiditis, which help distinguish it from subacute thyroiditis [64]. In the study by Lania et al. [42] all thyrotoxic patients did not have neck pain, but unfortunately, thyroid autoantibodies profile (i.e. TPOAb, TgAb and TRAb) was available in only nine patients and resulted negative. In hospitalized COVID-19 patients with clinical and radiological signs of pneumonia (i.e. patients enrolled in the study by Lania et al. [42]), neck pain associated with destructive thyrotoxicosis could missing because of the leucopenia. The low count of lymphocytes characterizing hospitalized COVID-19 patients could preclude the formation of giant cells (congregates of lymphocytes, histiocytes, and colloid) at the thyroid level with consequent absence of stretching of thyroid capsule and neck pain [44].

Also, it is important to note that 32% and 16% of overt thyrotoxic patients with COVID-19 also developed atrial fibrillation and thromboembolic events, respectively [42]. Moreover, it was noted that in thyrotoxic patients in-hospital mortality was higher and the duration of hospitalization was longer as compared to COVID-19 patients with normal thyroid function [42]. Therefore, thyrotoxicosis appears to be clinically relevant in COVID-19 patients, negatively impacting on their outcomes. One meta-analysis was published regarding the severity of COVID-19 in patients with pre-existing thyroid disease and it concluded that the presence of thyroid disease conferred a more severe degree of infection to COVID-19 [76]. However, some relevant limitations of the study do not allow us to generalize this finding: four of the eight included studies were published on “MedRxiv” (an online platform of non-peer-reviewed articles whose results should not be used for clinical medicine); one other included study was published in 2016 (so before the COVID-19 outbreak); it was not specified which kind of thyroid disorders was included in the term “thyroid disease” (i.e. hyperthyroidism, hypothyroidism, cancer) [76].

Lastly, two cases of COVID-19-related Graves’ disease were documented by Mateu-Salat et al. [46]: one with a previous history of Graves’ disease in remission for more than 30 years, and another with no history of thyroid disease. Thus, COVID-19 could be a trigger for new cases or relapses of Graves’ disease [8, 46].

### Hypothyroidism

Cases of COVID-19-related primary hypothyroidism has been reported in some studies [42, 44, 45]. Specifically, 5.2% (15/287) of patients in the study by Lania et al. [42] developed primary hypothyroidism, which was subclinical (i.e. FT3 and FT4 in the reference ranges) in about 90% of cases and overt in the remaining 10%. The authors [42] also found that in-hospital mortality of hypothyroid patients with COVID-19 was higher compared to that of COVID-19 patients with euthyroidism. Thus, similarly to thyrotoxicosis but maybe with lesser extent, hypothyroidism could negatively impact on outcome of COVID-19. In the study by Muller et al. [44] other two cases of primary hypothyroidism due to chronic autoimmune thyroiditis (CAT) were recorded among patients with COVID-19 admitted to high intensity of care units (HICUs). It seems that in both of these cases primary hypothyroidism developed during COVID-19 and persisted after discharge [44]. A case report of overt primary hypothyroidism due to CAT seven days after resolution of mild COVID-19 was reported by Tee et al. [45]. Therefore, there is some evidence that primary hypothyroidism could occur during or after COVID-19.

Central hypothyroidism is biochemically defined as low FT4 with inappropriately low/normal TSH [77]. Hormonal changes consistent with central hypothyroidism secondary to SARS-CoV-2 injury at hypothalamus or pituitary level of the HPT axis have been rarely described [37]. In the study by Chen et al. [37] central hypothyroidism could be diagnosed in 2–6% (one to three out of 50 patients) of patients hospitalized for non-mild COVID-19, who had low FT4 with low/normal TSH. Reversal of these hormonal changes occurred after recovery from COVID-19, a fact that highlights plausible acute/transitory effects of COVID-19 on HPT axis [37].

### Nonthyroidal illness syndrome

The nonthyroidal illness syndrome (NTIS) comprises a constellation of alterations in the central component of the HPT axis and changes in thyroid hormones (TH) metabolism in a variety of TH target organs [78]. NTIS can occur in several acute or chronic systemic diseases including cardiovascular, respiratory, infectious diseases and cancer [79, 80]. The most typical hormonal changes are low plasma T3, low or normal plasma T4, or elevated plasma reverse (rT3), in the presence of normal or slightly decreased TSH [79, 81]. The name “nonthyroidal illness syndrome” depends on the different hormonal profile compared to that of primary or secondary thyroid disorders [81]. Moreover, the synonym “sick euthyroid syndrome” is explained by the presence of normal TSH values in the presence of low T3 and at times also T4 concentrations, while the other synonym “low T3 syndrome” emphasizes that low T3 is the biochemical hallmark of this syndrome [81]. In an early phase of the systemic disease NTIS is thought to be an adaptive and protective state that conserves energy in an individual that is under stress and under macronutrient restriction [79, 81]. Instead, in the prolonged phase of critical illness when patients continue to depend on intensive medical care and parental nutrition, NTIS is associated to adverse outcomes, typically mortality [82]. Actually, critical patients who ultimately die have much lower plasma T4, T3, and TSH, and higher plasma rT3 than survivors [82]. Cytokines, released during illness, are considered a major determinant of NTIS since they affect a variety of genes involved in TH metabolism [78].

Therefore, it was conceivable that non-mild cases of COVID-19 could induce NTIS.

Indeed, severe and critical COVID-19 patients with NTIS were described in two studies [37, 44].

In particular, NTIS could underlie the hormonal changes of at least 30% (15/50) of hospitalized patients in Chen et al. [37]. And, as it has been already demonstrated for SARS [62], a significant positive correlation was found between the severity of COVID-19 and TSH and FT3 values [37]. Interestingly, without any thyroid replacement therapy, these hormonal changes normalized after recovery from COVID-19 [37]. In patients with COVID-19 pneumonia mean TSH and T3 (and FT3) values were found to be lower than control groups in two studies [37, 43], as a consequence of NTIS or a unique effect of SARS-CoV-2 on TSH secreting cells. However, also glucocorticoids could induce the observed decrease in TSH levels [37, 43].

Lastly, it is important to note that TSH and FT3 concentrations were significantly lower in deceased patients than in recovered patients with severe or critical confirmed COVID-19 [7]. The latter finding could be a fundamental clue of the value of low TSH and FT3 as predictors of poor outcome in severe and critical patients with COVID-19. In this scenario future studies should aim to support this evidence and explore the effect on outcome of treatment with specific drugs (i.e. hypothalamic releasing factors, triiodothyronine, thyroid hormone analogues) [53, 81, 83].

### Atypical thyroiditis

Atypical thyroiditis (AT) is a form of SAT, without neck pain, recognized in COVID-19 patients admitted to HICUs and in the context of NTIS [44]. Indeed, AT is biochemically characterized by low concentrations of TSH and FT3 along with normal or elevated concentrations of FT4, thus the synonym of “thyroxine thyrotoxicosis” [44]. As mentioned above, in non-mild COVID-19 patients the absence of neck pain could be due to lymphopenia [44]. Muller et al. [44] found that 15% (13/85) of COVID-19 patients admitted to HICUs had atypical thyroiditis. As opposed to classical SAT and COVID-19-related SAT, AT was more frequent in male patients, and this could be partially explained by the gender difference in the immune signatures associated to ACE2 at the thyroid level [50]. The development of AT might have contributed to the more critical conditions compared to patients admitted to HICUs in 2019 [44].

## Thyroid cancer patients in the time of COVID-19

The COVID-19 pandemic has deeply altered the conventional management of outpatient thyroid disorders as a consequence of social distancing policy, cut or closure of nonemergency health services, overburdened primary care, unavailability of diagnostic tolls and treatments [84]. In this context telemedicine could enhance specific aspects of thyroid care [84].

Data from China have listed thyroid cancer as a frequent diagnosis among patients admitted to hospitals with COVID-19 [85]. Moreover, during the COVID-19 pandemic, conventional strategies of care for thyroid nodule and cancer has been upset by the transmission risk of SARS-CoV-2 associated with in-person visits and diagnostic and therapeutical procedures. Some research teams have published their personal experiences on this topic to date [70–75, 85]. Differences in results and management of care likely reflect differences in local SARS-CoV-2 transmission rates and the ability of the health systems to manage.

### Experience of Endocrinology Divisions

Tsang et al. [70] reported that in 2020 72% of consultation were driven by telehealth, compared to only 4.9% of consultations in 2019. Moreover, they experienced a considerable decline in the number of fine-needle aspiration biopsies (FNAB) performed, about 60% compared to 2019 [70]. Treatment for high-risk thyroid cancers (including ^131^I ablation) was maintained [70]. Surgery for proven thyroid cancer has not been delayed, with similar numbers in the same months of 2019 and 2020 [70].

High patient-selection for FNAB and surgery was pursued by Smulever et al. [71] with consequent considerable decrease in these procedures of more than 98%, compared to the same period in 2019. ^131^I was postponed in all pending cancer cases with an intermediate risk of recurrence and only 3% of patients (7 of 223 cancer patients monitored since the COVID-19 pandemic began) with structural incomplete response underwent conventional pre-COVID-19 follow-up [71]. On the other hand, most of thyroid cancer patients were offered telemedicine or contacted to postpone the medical visit [71]. Smulever et al. [71] also reported their experience on advanced thyroid cancer: seven patients were unable to start multikinase inhibitors (MKIs) because of local health issues; the remaining 15 patients obtained frequent telehealth and in-person consultations, with resulting increase in telemedicine controls by 147%, and decrease in-office appointments to 35.5%. Moreover, only two patients underwent external beam radiation (EBR) therapy, performed on brain and pelvic metastasis [71]. Considering their intrinsic frailty and the negative impact on the immune system of both MKIs and EBR therapy, it is strongly suggested that patients under systemic treatment or recent EBR therapy be considered as a risk group for COVID-19 infection and poor outcomes [71].

One main issue was investigated by Falcone et al. [72] who analyzed the outbreak’s impact on emotional well-being and quality of life of patients with thyroid cancer. They found [72] that the Covid-19 pandemic is causing substantial emotional distress among thyroid cancer patients, regardless of their disease severity or current health-care needs. In addition, high scores of psychological distress were found among women and in patients < 65 years [72].

### Experience of surgery divisions

Three main reports, one from Italy, one from the kingdom of Jordan and one from China, documented the experience of Surgery Divisions on thyroid cancer management [73–75].

Elective surgery was allowed for patients included in the priority class (as defined on the basis of the Italian Plan for the Management of Waiting Lists 2019/2021) so that in a time period of four weeks 14 total thyroidectomies (plus lymph node dissection if needed) for thyroid cancer were performed [73]. However, this figure corresponded to about one-third of the usual amount of activity in the same time frame in the pre-COVID-19 era [73].

On the other hand, in the experience from Jordan, the restrictive measures during COVID-19 did not affect the safety and timely delivery of surgical care [74]. Indeed, between March 17 and May 20, 2020, all the 12 thyroid cancer surgeries were performed as scheduled without any kind of problems related to virus spread [74]. However, ^131^I treatment plans were altered considerably according to the outbreak: indeed, six patients opted for recombinant human TSH (rh-TSH) with additional extra personal cost of 1000 Jordanian Dinars (about 1400 US dollars) and one opted to delay ^131^I treatment until after lockdown [74].

In the largest study relative to the impact of COVID-19 on thyroid surgery to date [75], it was demonstrated the reduction of thyroid surgery volumes, also for thyroid malignancies, across the three phases of the COVID-19 outbreak (with the phase I indicating the time frame associated to the COVID-19 highest alert). The reduction of early stage cancer treatments, the decreased operative times and hospital stays, and the increased vocal cord paralysis (VCP) rate were other results of the study [75].

### Experience of nuclear medicine divisions

The international survey by Freudenberg et al. [86] demonstrated the worldwide precipitous decline in diagnostic and therapeutical nuclear medicine procedures. Specifically, they registered a decrease in thyroid studies by 67% and a reduction of radionuclide therapies of 45% on average [86]. It is of note that, in relation to staff health, 15% of respondents experienced COVID-19 within their own departments [86].

The study by Albano et al. [87] alerted Nuclear Medicine departments regarding the concrete possibility to identify through fluorine-18-deoxyglucose positron emission tomography/CT (^18^F-FDG-PET/CT) or single-photon emission computed tomography/CT (SPECT/CT) asymptomatic COVID-19 patients with signs of interstitial pneumonia. In particular, one of 12 patients undergoing ^131^I treatment for thyroid cancer showed incidental interstitial pneumonia at SPECT/CT [87].

## Conclusion

Theoretically, SARS-CoV-2 can involve any organ during the viraemic phase, and the thyroid and HPT axis involvement must be taken into consideration when facing with COVID-19. Table 2 contains a summary of findings regarding the relationship between thyroid and COVID-19.

**Table 2:** Summary of findings regarding the relationship between thyroid and COVID-19

ACE2 and TMPRSS2 expression levels are high in thyroid and more than in lungs [50]
Abnormal immune responses and cytokine storm associated to COVID-19 may induce thyroid gland inflammation [50, 54]
Two mechanisms (i.e. indirect and direct) might account for the changes in the thyroid gland and HPT axis [9–13]
COVID-19-related thyroid disorders could include thyrotoxicosis, hypothyroidism, nonthyroidal illness syndrome
COVID-19-related SAT is generally comparable to classical SAT and it can occur after or during COVID-19 [36]
Thyrotoxicosis in absence of neck pain is frequent in patients hospitalized for COVID-19 [42]
Low TSH and T3 and thyrotoxicosis appear to be predictors of poor outcome of patients hospitalized for COVID-19 [7]
Treatment plans for thyroid cancer are considerably changing in the direction of more teleconsultations and less diagnostic and therapeutical procedures [70–75]
Further research is necessary to explore the impact of the limitation of scheduled clinical activities on outcomes of thyroid cancer patients and whether thyroid cancer (or treatment-specific factors) increase vulnerability to COVID-19

ACE2, Angiotensin-converting–enzyme 2; TMPRSS2, transmembrane protease serine 2;

HPT, hypothalamic–pituitary–thyroid; SAT, subacute thyroiditis

Preclinical and clinical studies find compelling evidence that the thyroid gland can be a target organ of COVID-19. The involvement of the thyroid gland (and HPT axis) by COVID-19 manifests as thyroid disorders and hormonal changes. The severity of COVID-19 seems to be the main determinant of the type of alteration that dominates thyroid injury. Specifically, while destructive thyrotoxicosis associated with neck pain (i.e. classical subacute thyroiditis) mainly occurs during or soon after mild COVID-19, thyrotoxicosis without neck pain (possibly in the context of the nonthyroidal illness syndrome) could characterize more severe and critical cases of COVID-19 pneumonia. As it is known outside the COVID-19 scenario, some clues of the hormonal changes (i.e. low T3 and TSH concentrations) and overt thyrotoxicosis to be regarded as predictors of poor outcome (i.e. longer length of hospital stay and higher mortality) of COVID-19 are already emerging. The collected studies on “thyroid and COVID-19” suggest for the monitoring of thyroid function tests during acute illness as well as during convalescence of SARS-CoV-2 with the possibility of therapy as indicated. However, data on therapies of thyrotoxicosis and the nonthyroidal illness syndrome in hospitalized cases of COVID-19 are lacking.

Treatment plans for thyroid cancer are considerably changing during the COVID-19 pandemic in the direction of more teleconsultations and less diagnostic and therapeutical procedures. Further research would be necessary to explore the effects of the limitation of certain scheduled clinical activities on outcomes for untreated or under-diagnosed thyroid cancer patients and whether thyroid cancer (or treatment-specific factors) increase vulnerability to COVID-19.

Nobody knows how long the ongoing COVID-19 pandemic will be lasting, but in the next future, it is expected heavy demand for healthcare non-COVID-19 services.

The current review includes findings that could be changed soon by new results on the topic, considering the rapidity and the huge amount of worldwide research on COVID-19.

## Abbreviations

ACE2: angiotensin-converting–enzyme 2
AITD: autoimmune thyroid diseases
ARDS: acute respiratory distress syndrome
AT: atypical thyroiditis
CAT: chronic autoimmune thyroiditis
COVID-19: Coronavirus disease 2019
CRP: C-reactive protein
EBR: external beam radiation
FNAB: fine-needle aspiration biopsies
FT3: free T3
FT4: free T4
HICU: high intensity of care unit
HPT: hypothalamic–pituitary–thyroid
MKI: multikinase inhibitors
NK: natural killer
NSAID: non-steroidal anti-inflammatory drug
NTIS: nonthyroidal illness syndrome
ONFH: osteonecrosis of the femoral head
ORs: olfactory receptors
rh-TSH: recombinant human TSH
SARS: severe acute respiratory syndrome
SARS-CoV-2: severe acute respiratory syndrome coronavirus 2
SAT: subacute thyroiditis
SPECT/CT: single-photon emission computed tomography/CT
TH: thyroid hormones
TLR: toll-like receptor
TMPRSS2: transmembrane protease serine 2
TPOAb: thyroperoxidase antibodies
TRAb: TSH receptor antibodies
VCP: vocal cord paralysis
18F FDG-PET/CT: fluorine-18-deoxyglucose positron emission tomography/CT
